# The Dark Side of Radiotherapy: Experimental Evidence, Biological Mechanisms, and Preventive Strategies for Radiation-Induced Tumor-Promoting Niches

**DOI:** 10.3390/biomedicines14102292

**Published:** 2026-10-09

**Authors:** Yong June Choi

**Affiliations:** Research Institute of Pharmaceutical Sciences, College of Pharmacy, Seoul National University, Seoul 08826, Republic of Korea; cyjune112@snu.ac.kr

**Keywords:** radiotherapy, radiation-induced tumor-promoting niches

## Abstract

Radiotherapy (RT) is an essential treatment modality for solid tumors and achieves tumor control primarily through radiation-induced cytotoxicity. However, its biological effects extend beyond direct tumor-cell killing. Radiation inevitably affects surrounding normal and tumor-adjacent tissues, inducing inflammatory, stromal, extracellular-matrix, vascular, and cellular remodeling that can persist after treatment. Accumulating experimental evidence indicates that these tissue responses can generate microenvironments permissive to the survival and regrowth of residual tumor cells, local reseeding, and metastatic colonization. This review examines radiation-induced tumor-promoting niches as an unintended consequence of tissue injury and repair. Experimental and clinical evidence supporting their existence is summarized, followed by discussion of the major biological processes underlying their formation, including immune remodeling, fibroblast activation and extracellular-matrix reorganization, vascular injury and hypoxia-driven reparative vasculogenesis, and cellular senescence. Emerging strategies to prevent or attenuate these niches are also discussed, including limiting normal-tissue radiation exposure and selectively targeting persistent inflammatory and stromal responses, post-radiation vasculogenesis, and senescent cells. Although direct causal evidence remains predominantly preclinical and the clinical significance of these responses remains to be established, understanding how irradiated tissues evolve after treatment may reveal opportunities to preserve the therapeutic benefits of RT while minimizing long-term tissue conditions that favor tumor recurrence or metastatic progression.

## 1. Introduction: Radiotherapy Beyond Tumor-Cell Killing

Radiotherapy (RT) is one of the most effective and widely utilized treatment modalities for solid malignancies and remains a cornerstone of modern cancer therapy [1,2]. Approximately 50–60% of patients with cancer receive RT during the course of their disease, either alone or in combination with surgery, chemotherapy, targeted therapy, or immunotherapy [1,2,3]. Depending on the clinical setting, RT is administered with curative, neoadjuvant, adjuvant, or palliative intent, contributing substantially to improved local tumor control, organ preservation, symptom relief, and overall survival across a broad spectrum of malignancies, including breast, lung, prostate, head and neck, cervical, and gastrointestinal cancers [4,5,6]. The therapeutic efficacy of RT primarily relies on its ability to induce irreversible DNA damage in tumor cells through both direct ionization and indirect generation of reactive oxygen species (ROS), ultimately leading to apoptosis, mitotic catastrophe, senescence, or other forms of cell death [7,8]. In addition to direct cytotoxicity, RT can enhance anti-tumor immunity by promoting immunogenic cell death, increasing tumor antigen release, and facilitating immune cell activation [9]. Advances in radiation delivery technologies, including intensity-modulated radiotherapy (IMRT), image-guided radiotherapy (IGRT), stereotactic body radiotherapy (SBRT), proton therapy, and adaptive RT, have further improved the therapeutic ratio by maximizing tumor dose while minimizing irradiation of surrounding normal tissues [10,11,12]. Collectively, these developments have established RT as an indispensable component of multidisciplinary cancer treatment.

Despite these remarkable therapeutic benefits, ionizing radiation cannot selectively target malignant cells without affecting neighboring normal tissues. Even with highly conformal treatment techniques, epithelial cells, endothelial cells, fibroblasts, immune cells, and extracellular-matrix (ECM) components located within or adjacent to the irradiated field inevitably receive radiation exposure [13,14,15]. Consequently, RT induces a broad spectrum of biological responses in normal tissues that extend well beyond the elimination of tumor cells. The consequences of normal-tissue irradiation have traditionally been considered predominantly in the context of radiation-induced toxicities, including dermatitis, mucositis, pneumonitis, enteritis, vascular injury, and fibrosis [16,17,18]. However, radiation injury can initiate tissue-remodeling processes that persist long after treatment, progressively altering the composition and function of the local tissue microenvironment [16,17,18]. Accumulating experimental evidence indicates that, under certain conditions, these persistent tissue alterations can generate microenvironments that support tumor recurrence, metastatic colonization, and therapeutic resistance [19,20,21,22,23,24,25,26,27,28,29]. These observations broaden the current understanding of the biological consequences of RT, indicating that radiation-induced tissue remodeling may influence not only normal-tissue toxicity but also subsequent cancer progression.

Radiation-induced tissue remodeling involves coordinated responses across multiple cellular and structural compartments [14]. Persistent inflammatory responses, stromal activation, vascular alterations, extracellular-matrix remodeling, and chronic wound-healing programs may reshape the local tissue environment long after the completion of radiation treatment [14]. These adaptive responses initially function to restore tissue integrity and maintain tissue homeostasis following radiation-induced injury [14]. However, when tissue repair becomes excessive, prolonged, or dysregulated, the resulting microenvironment may differ substantially from its pre-irradiation state and acquire biological properties that inadvertently favor malignant progression [14]. An increasing number of experimental studies have demonstrated that previously irradiated tissues can become permissive sites for tumor regrowth, local recurrence, or metastatic colonization [19,20,21,22,23,24,25,26,27,28,29]. Consistent with these experimental findings, clinical studies have also suggested associations between RT and unfavorable oncologic outcomes in certain clinical contexts [30,31,32,33]. Although RT effectively eradicates the majority of tumor cells, the remodeled tissue microenvironment generated during tissue repair may provide survival advantages for therapy-resistant cancer cells or disseminated tumor cells that subsequently colonize irradiated organs. These observations suggest that the biological consequences of RT extend beyond direct tumor-cell killing and include long-lasting alterations of the surrounding host tissues that may influence future tumor behavior.

In this Review, the term “radiation-induced tumor-promoting niche” is used to describe a persistently remodeled tissue microenvironment in which radiation-induced host responses acquire properties that support subsequent tumor survival, regrowth, invasion, or metastatic colonization. Rather than being driven by a single cell type or signaling pathway, these niches arise through coordinated alterations involving inflammatory responses, stromal remodeling, vascular dysfunction, extracellular-matrix reorganization, immune regulation, and tissue repair. The following sections summarize current evidence supporting the existence of radiation-induced tumor-promoting niches and discuss the cellular mechanisms, molecular pathways, and emerging preventive strategies involved in their formation.

## 2. Evidence That Radiotherapy Can Generate Tumor-Promoting Niches

Evidence that RT can generate tumor-promoting tissue environments has emerged from several complementary experimental settings. The most direct studies irradiated normal tissues before introducing non-irradiated tumor cells, thereby separating the effects of radiation on host tissues from its direct effects on malignant cells. Other studies examined tumors that developed or recurred within previously irradiated tissue beds, as well as residual tumors exposed to subcurative radiation. Collectively, preclinical models provide direct evidence that irradiation of host tissues can create conditions that favor malignant progression. The principal preclinical models supporting these observations are summarized in Table 1. Clinical evidence is considerably less direct, because the possibility that irradiated tissues subsequently acquire tumor-promoting properties has rarely been examined as a predefined clinical endpoint. Nevertheless, several clinical and translational observations have reported patterns potentially relevant to this concept. Accordingly, the strongest causal evidence currently derives from preclinical models that isolate host-tissue effects from direct tumor-cell irradiation or experimentally modify the implicated host response, whereas the available clinical evidence remains largely associative and hypothesis-generating rather than direct validation of the proposed niche concept in patients.

### 2.1. Experimental Evidence from Pre-Irradiated Normal Tissues

One of the clearest approaches for demonstrating a radiation-induced tumor-promoting niche is to irradiate a normal organ first and introduce tumor cells only after the radiation exposure. Because the tumor cells themselves are not irradiated, any change in their subsequent behavior can be attributed primarily to alterations in the host tissue.

Early evidence for this concept was obtained in the mammary gland. Barcellos-Hoff and Ravani irradiated the mammary glands of mice and subsequently transplanted non-irradiated mammary epithelial cells into irradiated or control mammary tissue [19]. Cells transplanted into the irradiated mammary stroma showed a greater frequency of tumor formation than those transplanted into non-irradiated tissue [19]. Because the transplanted epithelial cells were not themselves exposed to radiation, the study demonstrated that radiation-induced alterations in the host stroma could promote the expression of pre-existing tumorigenic potential [19]. These findings provided early direct evidence that irradiated normal tissue can actively influence subsequent tumor development rather than serving merely as a passively damaged substrate. Bouchard et al. investigated whether irradiation of the mammary gland could alter the local breast tumor microenvironment and promote metastatic dissemination in a triple-negative breast cancer model [20]. BALB/c mice received localized irradiation to the third mammary gland, followed by implantation of breast cancer cells into the irradiated mammary fat pad. Pre-irradiation of the mammary gland significantly increased local tumor-cell invasion, the number of circulating tumor cells, and subsequent lung metastases [20]. Importantly, these radiation-induced effects were abolished when MT1-MMP expression was downregulated in tumor cells, demonstrating that radiation-induced remodeling of the mammary microenvironment cooperated with tumor-cell invasive machinery to facilitate metastatic dissemination [20].

More recent studies have extended this concept to metastatic colonization of the lung. Feys and colleagues investigated radiation-induced pulmonary injury using breast cancer models and found that prior lung irradiation enhanced several early steps of metastatic colonization [22]. Irradiated lungs showed increased adhesion and retention of intravenously introduced breast cancer cells, followed by increased pulmonary metastatic burden [22]. The study implicated stromal cell-derived factor 1, also known as CXCL12, and its receptor CXCR4 in the enhanced interaction between breast cancer cells and irradiated pulmonary tissue [22]. Pharmacological interference with CXCR4 signaling reduced the radiation-associated metastatic phenotype [22]. These findings suggested that radiation-induced lung injury can increase the retention of circulating tumor cells within the lung and generate chemokine signals that facilitate subsequent metastatic colonization. Nolan and colleagues irradiated healthy mouse lungs before experimentally inducing metastasis and observed a marked increase in the growth of subsequently arriving cancer cells [24]. Radiation induced a rapid neutrophil-dominated injury response in the lung, and locally activated neutrophils induced regenerative Notch signaling in the irradiated tissue, thereby promoting a tumor-supportive pulmonary microenvironment [24]. This altered tissue state increased the stem-like properties of colonizing cancer cells. Pharmacological inhibition of neutrophil degranulation suppressed neutrophil-dependent Notch activation and significantly reduced radiation-enhanced metastatic growth, providing functional evidence that the neutrophil response contributed directly to the formation of a permissive metastatic niche [24]. Choi and colleagues recently established radiation-induced lung and liver injury models by exposing the thorax or liver of mice to localized irradiation before introducing metastatic tumor cells intravenously or via intrasplenic injection [21]. Prior thoracic irradiation significantly increased pulmonary engraftment of both melanoma and breast cancer cells and reduced survival despite the absence of overt clinical deterioration caused by radiation alone [21]. At the time tumor cells were introduced, conventional imaging did not reveal severe lung injury; nevertheless, molecular analyses detected alveolar collapse, collagen accumulation, fibrotic gene expression, macrophage infiltration, and myofibroblast activation [21]. Depletion of macrophages reduced radiation-enhanced pulmonary tumor-cell engraftment, whereas inhibition of SRC-family kinases with dasatinib or the selective SRC/YES1 inhibitor suppressed early fibrotic remodeling and prevented metastatic colonization [21]. Additionally, localized irradiation of the liver similarly enhanced hepatic metastasis following intrasplenic injection of colorectal cancer cells [21], suggesting that radiation-induced tissue remodeling can generate permissive metastatic niches in multiple organs and is not restricted to the lung.

Additional evidence supporting this concept has also been provided by classical tumor-bed studies. Milas and colleagues irradiated prospective tumor beds before implantation of several murine sarcoma and carcinoma models [23]. Although tumors growing within irradiated tissues generally exhibited delayed primary growth, consistent with impaired support from the irradiated tumor bed, they also produced more distant metastases than tumors growing in non-irradiated tissues [23]. These findings suggested that radiation-induced injury to the host tissue could uncouple local tumor growth from metastatic potential, indicating that reduced primary tumor growth does not necessarily reflect diminished malignant behavior. Rofstad and colleagues further investigated the biological basis of this phenomenon using two human melanoma xenograft models implanted into pre-irradiated tissue [25]. Tumors developing within irradiated tissue beds exhibited increased metastatic dissemination despite slower local growth [25]. Mechanistically, pre-irradiated tissues displayed impaired vascular development, resulting in pronounced intratumoral hypoxia [25]. This hypoxic environment promoted expression of metastasis-associated molecules, including IL-8 in the pulmonary metastatic model and urokinase-type plasminogen activator receptor (uPAR) in the lymphatic metastatic model [25]. Neutralization of IL-8 or uPAR significantly reduced metastatic dissemination, demonstrating that radiation-induced stromal remodeling can indirectly promote metastatic progression by generating a hypoxic microenvironment that drives prometastatic gene expression [25].

Collectively, these experimental studies demonstrate that radiation-induced tumor-promoting remodeling is not restricted to a single tissue or experimental model and has been observed across several organ systems. Across these models, tumor-promoting effects have been associated with distinct but overlapping features, including inflammatory-cell recruitment, extracellular-matrix remodeling, vascular dysfunction, hypoxia, and fibrotic changes, which can enhance tumor-cell retention, invasion, metastatic colonization, or dissemination [19,20,21,22,23,24,25]. Together, these studies provide direct experimental evidence that radiation can generate tumor-promoting niches independently of its direct effects on tumor cells.

### 2.2. Radiation-Induced Tissue Responses Support Tumor Recurrence and Local Reseeding

In addition to creating permissive environments before tumor-cell arrival, radiation-induced tissue responses can also support the recurrence of residual tumors or the recruitment of circulating tumor cells to previously irradiated sites. These models differ from the pre-irradiated normal-tissue models described above because radiation is delivered in the context of an established tumor or after primary tumor development. In these experimental settings, tumor recurrence or reseeding cannot be explained solely by the intrinsic properties of surviving cancer cells. Radiation-induced vascular injury, inflammatory-cell recruitment, and host tissue-repair responses can also provide microenvironmental support for residual or newly recruited tumor cells to re-establish growth.

Kioi and colleagues investigated how residual glioblastoma cells restore their vascular supply after irradiation [26]. Human glioblastoma cells were implanted intracranially in mice, and the established tumors were subsequently irradiated. Radiation initially reduced tumor burden and damaged the local vasculature, but surviving tumor cells eventually resumed growth [26]. Following irradiation, vascular recovery and tumor regrowth became strongly dependent on recruitment of bone marrow-derived cells and vasculogenesis rather than conventional angiogenesis from surviving local vessels [26]. Radiation-induced vascular injury increased intratumoral hypoxia and HIF-1 activity, leading to increased expression of stromal cell-derived factor 1 (SDF-1/CXCL12). CXCL12 subsequently recruited predominantly CD11b-positive myelomonocytic cells from the bone marrow into the irradiated tumor [26]. These recruited cells contributed to the restoration of functional tumor vasculature and supported the regrowth of surviving glioblastoma cells [26]. Pharmacological inhibition of HIF-1 or disruption of CXCL12–CXCR4 signaling, including CXCR4-neutralizing antibodies, reduced myeloid-cell recruitment, impaired vascular restoration, and prevented tumor recurrence [26]. Rafat and colleagues identified a distinct mechanism through which irradiated tissues may contribute to local recurrence, the recruitment of circulating tumor cells back to the irradiated site [27]. Using breast cancer models, the investigators showed that local irradiation altered normal tissues before detectable tumor-cell infiltration and induced the accumulation of macrophages within the irradiated field [27]. Circulating tumor cells were subsequently recruited more efficiently to irradiated than to non-irradiated tissues, particularly in mice lacking CD8-positive T-cell-mediated immune surveillance [27]. Macrophage accumulation preceded tumor-cell recruitment, suggesting that these cells contributed to the establishment of a tissue environment permissive to local reseeding [27]. Treatment with the CCR5 antagonist reduced macrophage infiltration and decreased the recruitment of circulating tumor cells to irradiated tissues [27]. The study therefore indicated that, in this experimental setting, local tumor recurrence after irradiation need not arise exclusively from cancer cells surviving within the original tumor bed. Instead, circulating tumor cells may return to and recolonize radiation-remodeled tissues [27]. These studies demonstrate that radiation-induced tumor-promoting effects can operate through distinct mechanisms after treatment. In these experimental models, recurrence or reseeding was influenced not only by tumor-cell-intrinsic properties but also by radiation-induced changes in the host microenvironment. In selected experimental contexts, the irradiated host tissue actively contributes to the re-establishment of tumor growth through vascular repair, inflammatory-cell recruitment, and immune dysregulation [26,27].

### 2.3. Irradiated Tumor and Stromal Cells Acquire Tumor-Promoting Properties

Although the most direct evidence for radiation-induced tumor-promoting niches comes from host-tissue irradiation, radiation can also alter tumor cells and non-malignant stromal cells that survive within the irradiated tumor bed. These surviving cells do not necessarily remain biologically inactive after treatment. Instead, experimental studies indicate that they can acquire properties that enhance the invasion, dissemination, or metastatic growth of neighboring cancer cells. These findings broaden the concept by showing that both host-tissue remodeling and functional changes in surviving cells within the irradiated field can contribute to a post-radiation tumor-supportive microenvironment.

Shen and colleagues examined whether irradiated tumor cells could influence the behavior of neighboring non-irradiated cancer cells [28]. Irradiation of lung adenocarcinoma cells increased the secretion and enzymatic activity of lysyl oxidase (LOX) in a radiation-dose-dependent manner [28]. Conditioned medium collected from irradiated tumor cells increased the invasiveness of non-irradiated tumor cells, whereas inhibition or knockdown of LOX in the irradiated cells reduced this invasion-promoting effect [28]. The investigators also found increased LOX expression within irradiated tumor xenografts and increased circulating LOX activity following local tumor irradiation in mice [28]. These findings demonstrated that tumor cells surviving irradiation can release biologically active factors that promote the invasive behavior of neighboring cancer cells. Thus, the consequences of radiation exposure may extend beyond the directly irradiated tumor-cell population and influence non-irradiated cells through changes in the tumor secretome. Radiation can also modify stromal cells in a manner that increases their capacity to promote malignant progression. Li and colleagues investigated carcinoma-associated fibroblasts in pancreatic cancer and showed that irradiated fibroblasts enhanced pancreatic cancer-cell migration and invasion more strongly than non-irradiated fibroblasts [29]. Pancreatic cancer cells exposed to irradiated fibroblasts or their conditioned medium acquired features consistent with epithelial-to-mesenchymal transition (EMT), including reduced epithelial characteristics and increased mesenchymal and invasive behavior [29]. The study further showed that irradiation increased CXCL12 production by carcinoma-associated fibroblasts and that inhibition of the CXCL12–CXCR4 pathway reduced the fibroblast-mediated migration, invasion, and EMT of pancreatic cancer cells [29]. In an in vivo experimental metastasis model, pancreatic cancer cells preconditioned with irradiated fibroblasts showed increased metastatic colonization of the lung compared with cells exposed to non-irradiated fibroblasts [29]. These findings demonstrated that radiation can enhance the tumor-promoting activity of fibroblasts and thereby increase the invasive and metastatic potential of nearby pancreatic cancer cells.

Together, these studies demonstrate that radiation-induced tumor-promoting effects are not limited to remodeling of previously normal tissues. Tumor cells and stromal fibroblasts that survive irradiation can themselves acquire functional properties that promote the invasion and metastatic behavior of neighboring cancer cells. Irradiated tumor cells can release invasion-promoting factors, whereas irradiated fibroblasts can provide enhanced stromal support for tumor-cell migration, EMT, and metastatic colonization. These observations provide complementary evidence that surviving cellular components within the irradiated tumor microenvironment can actively influence subsequent tumor behavior.

### 2.4. Clinical and Translational Observations Relevant to the Hypothesis

Clinical evidence for radiation-induced tumor-promoting niches remains substantially less developed than the corresponding experimental evidence. Importantly, the clinical studies discussed below were not designed to directly establish whether radiation-induced remodeling of normal or tumor-adjacent tissues causally promotes subsequent tumor recurrence or metastatic progression. Instead, these studies primarily evaluated clinical outcomes such as recurrence, metastatic patterns, disease-free survival, or overall survival. Therefore, their relevance to the proposed niche concept must be interpreted according to study design, patient selection, treatment era, statistical adjustment, and alternative explanations for the observed outcomes. The principal quantitative findings and methodological limitations of these studies are summarized in Table 2.

One of the earliest clinical observations was reported by Dao and Kovaric [30]. The investigators compared women with breast cancer treated by mastectomy alone with those who received mastectomy followed by postoperative irradiation and reported a higher incidence of pulmonary and skin metastases in the irradiated group [30]. This observation raised the possibility that postoperative radiation exposure might influence subsequent metastatic patterns [30]. However, because the study predated modern staging, systemic therapy, and radiation-planning methods, the contribution of radiation itself to the observed metastatic pattern could not be clearly determined.

Importantly, several randomized trials and subsequent meta-analyses have reported that RT may not always translate into improved overall survival in every clinical setting [31,32]. A representative example is the individual-patient-data meta-analysis performed by the PORT Meta-analysis Trialists Group, which evaluated postoperative RT following complete resection of non-small-cell lung cancer [31]. The original analysis demonstrated that postoperative RT was associated with a significant adverse effect on overall survival (hazard ratio, 1.21; 95% CI, 1.08–1.34), corresponding to an absolute reduction in 2-year overall survival from 55% to 48%, with the greatest detrimental effect observed in patients with stage I–II or N0–N1 disease [31]. An updated analysis by the same collaborative group subsequently confirmed that postoperative RT remained associated with an increased risk of death and poorer recurrence-free outcomes, particularly in patients with early-stage disease [32]. However, because these trials were conducted before the widespread implementation of contemporary radiation-planning techniques and normal-tissue dose constraints, treatment-related cardiopulmonary toxicity has been considered a major contributor to the observed survival disadvantage. Thus, although these studies do not establish that radiation-induced tissue remodeling contributed directly to the observed survival disadvantage, the possibility that persistent host-tissue remodeling may have influenced clinical outcomes in at least a subset of patients cannot be excluded.

Comparative studies of early-stage non-small-cell lung cancer have reported less favorable local recurrence outcomes after SBRT than after surgical resection [33]. In a propensity score-matched analysis, Sebastian and colleagues found that SBRT was associated with a significantly higher risk of local recurrence than lobectomy (hazard ratio, 6.80; 95% CI, 1.92–24.10), whereas distant recurrence did not differ significantly between the two treatment groups [33]. Because patients treated with SBRT were generally older, medically less fit, and unsuitable for surgery, these findings cannot be interpreted as direct evidence that radiation-induced tissue remodeling promoted recurrence. However, they leave open the possibility that, in addition to residual disease, intrinsic tumor biology, and patient-related factors, radiation-induced alterations within the irradiated tissue may have contributed to the increased local recurrence observed after SBRT.

Not all clinical studies have supported an association between prior RT and more aggressive tumor behavior after recurrence. Woodward and colleagues analyzed women who developed locoregional breast cancer recurrence after mastectomy and compared clinical outcomes according to previous receipt of postmastectomy RT [34]. Although patients who had previously received RT initially appeared to have poorer outcomes, they also had more advanced baseline disease and were more likely to have distant metastases before or at the time of recurrence [34]. After adjustment for these clinical differences, prior RT was not associated with reduced survival or a more aggressive recurrence phenotype among patients with isolated locoregional recurrence [34]. These findings underscore the difficulty of separating radiation-associated tissue effects from baseline disease characteristics and other determinants of recurrence in clinical cohorts.

Taken together, the available clinical evidence suggests that the oncologic consequences of RT extend beyond its direct effects on tumor eradication and may, under certain clinical contexts, be influenced by biological changes occurring within irradiated tissues. Although current clinical studies do not establish a causal relationship between radiation-induced tissue remodeling and tumor recurrence or metastasis, several historical observations, comparative outcome studies, and translational analyses support the possibility that persistent alterations within irradiated tissues may contribute to unfavorable clinical outcomes in at least a subset of patients [30,31,32,33].

## 3. Biological Processes Underlying Radiation-Induced Tumor-Promoting Niches

Radiation-induced tumor-promoting niches can involve coordinated alterations in immune cells, stromal cells, vascular networks, and extracellular-matrix components [7,8,14]. Ionizing radiation induces cell death, oxidative stress, vascular injury, and disruption of normal tissue architecture [7,8,14]. These events initiate inflammatory and reparative programs intended to remove damaged cells and restore tissue integrity. When the injury is limited and repair proceeds normally, the tissue may return toward homeostasis [16]. By contrast, persistent injury, incomplete vascular recovery, sustained immune-cell recruitment, and prolonged fibroblast activation can maintain a remodeled tissue state [16]. The resulting microenvironment may support the retention, survival, invasion, or regrowth of residual and disseminated tumor cells. These processes are highly interconnected and should be viewed as components of an evolving tissue response rather than as independent mechanisms.

### 3.1. Immune Remodeling

Radiation-induced immune remodeling begins immediately after tissue injury and evolves through a coordinated sequence of inflammatory and reparative responses [16]. Following irradiation, damaged epithelial, endothelial, stromal, and tumor cells release damage-associated molecular patterns (DAMPs), reactive oxygen species (ROS), cytokines, and chemokines that rapidly activate innate immunity and recruit circulating leukocytes [16,35]. These early inflammatory responses contribute to the clearance of damaged cells and initiation of tissue repair. Under physiological conditions, inflammation gradually resolves as tissue integrity and immune homeostasis are restored [36,37]. However, when tissue damage persists or repair becomes dysregulated, inflammatory responses fail to resolve completely and progressively shift toward a chronic wound-healing state [18,38]. Rather than simply reflecting prolonged inflammation or immunosuppression, persistent immune remodeling may contribute to a microenvironment that supports tumor survival, recurrence, or metastatic colonization.

#### 3.1.1. Neutrophils

Neutrophils are among the first immune cells recruited to irradiated tissues and serve as key initiators of the early wound-healing response [24,39]. Following radiation-induced tissue injury, neutrophils remove damaged cells through phagocytosis and degranulation while releasing proteases, reactive oxygen species, and regenerative mediators that promote epithelial repair [24]. These functions can contribute to restoration of tissue integrity. However, radiation-recruited neutrophils can also alter regenerative signaling within injured tissues, thereby creating conditions permissive for subsequent tumor-cell colonization [24]. Consistent with these biological functions, experimental studies have demonstrated that radiation-induced neutrophils promote metastatic colonization of previously irradiated lungs by activating regenerative Notch signaling and enhancing stem-like properties of disseminated tumor cells [24]. Importantly, radiation-induced neutrophil responses can actively contribute to the establishment of a tumor-permissive tissue environment rather than merely reflecting tissue inflammation [24].

#### 3.1.2. Macrophages

Macrophages are another major immune population involved in inflammatory and reparative responses within irradiated tissues [21,27]. Through efferocytosis, macrophages remove apoptotic cells and subsequently coordinate fibroblast activation, extracellular-matrix remodeling, angiogenesis, and vascular repair by producing cytokines, growth factors, and matrix-remodeling enzymes [40,41]. These repair programs are essential for normal tissue regeneration but may become maladaptive when macrophage activation persists. Rather than existing as discrete M1 or M2 populations, radiation-associated macrophages display substantial functional plasticity and integrate signals from hypoxia, stromal cells, extracellular matrix, and adaptive immune cells [41]. Experimental studies demonstrate that radiation-induced macrophage accumulation can contribute directly to tumor-promoting tissue remodeling [21,27]. Macrophage depletion reduces tumor-cell engraftment in pre-irradiated lungs, and inhibition of macrophage recruitment reduces the recruitment of circulating tumor cells to irradiated tissues [21,27]. Collectively, these findings support a role for macrophages in linking radiation-induced immune responses with stromal remodeling and subsequent tumor-cell recruitment or growth.

#### 3.1.3. Adaptive Immune Surveillance

Adaptive immunity can influence the balance between antitumor immune surveillance and tumor-supportive inflammatory responses within irradiated tissues [16]. Radiation can enhance antigen presentation and tumor-specific CD8^+^ T-cell activation through immunogenic cell death, thereby contributing to antitumor immunity [9]. Conversely, RT can also suppress adaptive immune surveillance. Lymphocytes are highly radiosensitive, and treatment-related lymphopenia can persist after RT, potentially compromising effective T-cell-mediated tumor surveillance [42]. Loss of effective immune surveillance not only limits elimination of residual tumor cells but also permits prolonged myeloid-driven repair responses that favor tumor-cell recruitment and survival within irradiated tissues. Experimental evidence supports this concept by demonstrating that impaired CD8^+^ T-cell surveillance facilitates macrophage-dependent recruitment of circulating tumor cells into irradiated tissues, linking defective adaptive immunity to the establishment of tumor-promoting niches [27].

Taken together, radiation-induced immune remodeling involves temporally overlapping and interacting innate and adaptive immune responses rather than independent activation of individual immune-cell populations. Neutrophils participate prominently in the early inflammatory response, macrophages contribute to subsequent inflammatory and reparative remodeling, and adaptive immune surveillance can influence the recruitment and persistence of tumor cells within irradiated tissues. When inflammatory and reparative responses persist, interactions among immune cells, stromal cells, vascular compartments, and the extracellular matrix may contribute to the establishment of a tumor-promoting tissue environment. These interactions provide a mechanistic link between radiation-induced immune responses and the stromal and fibrotic remodeling discussed in the following section.

### 3.2. Stromal Remodeling: From Tissue Repair to Fibrosis

Fibrosis is traditionally regarded as a late manifestation of radiation-induced normal-tissue toxicity [18]. However, increasing evidence suggests that fibrotic remodeling is not simply the accumulation of scar tissue but a dynamic biological process capable of reshaping the tumor microenvironment [18,21,43]. Following radiation injury, fibroblasts, immune cells, endothelial cells, and extracellular-matrix components collectively participate in tissue repair [18,21,43]. Although these responses are essential for restoration of tissue integrity, persistent stromal activation can progressively alter the biochemical, structural, and mechanical properties of irradiated tissues, potentially creating conditions favorable for subsequent tumor growth or metastatic colonization.

#### 3.2.1. Fibroblast Activation

Fibroblasts are the principal stromal cells responsible for maintaining extracellular-matrix homeostasis and coordinating tissue repair after injury [18]. Radiation can promote fibroblast activation and phenotypic remodeling both directly through radiation-induced cellular stress and indirectly through cytokines released from immune and epithelial cells [18]. Activated fibroblasts can differentiate into myofibroblasts and increase the production of collagen, fibronectin, and other extracellular-matrix proteins together with multiple growth factors and chemokines [18]. Under physiological conditions, these responses resolve once tissue repair is completed. However, persistent fibroblast activation maintains a chronically remodeled stromal environment capable of supporting tumor-cell survival and dissemination [21,44]. Experimental evidence supports this concept. Transplantation studies demonstrated that non-irradiated mammary epithelial cells acquire significantly greater tumorigenic potential when placed into previously irradiated mammary stroma, indicating that alterations in the irradiated host stroma can enhance tumor development without direct irradiation of the transplanted epithelial cells [19]. Likewise, irradiated cancer-associated fibroblasts exhibit enhanced secretion of CXCL12 and promote migration, invasion, and EMT of neighboring pancreatic cancer cells through CXCR4 signaling, demonstrating that stromal cells surviving radiation can actively modify tumor behavior [29].

#### 3.2.2. Extracellular-Matrix Remodeling (ECM)

Radiation-induced stromal activation is accompanied by extensive remodeling of the ECM [45]. Beyond providing structural support, the ECM regulates cell adhesion, migration, mechanotransduction, and the bioavailability of growth factors [46]. Following radiation injury, ECM remodeling involves alterations in both matrix composition and organization, including increased collagen deposition, collagen crosslinking, and proteolytic remodeling [45]. Collagen crosslinking is primarily mediated by LOX, which increases matrix stability and stiffness, whereas matrix metalloproteinases (MMPs) contribute to ECM degradation and reorganization [46,47,48]. These processes are normal components of tissue repair but can become persistent during chronic remodeling of irradiated tissues. Experimental studies support the involvement of these pathways following radiation. Ionizing radiation increases the secretion of enzymatically active LOX from surviving tumor cells, and conditioned medium from irradiated cells enhances the invasive capacity of non-irradiated cancer cells in a LOX-dependent manner [28]. In a separate pre-irradiated mammary-gland model, suppression of tumor-cell MT1-MMP abolished radiation-enhanced local invasion and pulmonary metastasis, demonstrating a functional requirement for MT1-MMP-dependent matrix remodeling in this model [20]. These findings support the concept that radiation-induced ECM remodeling may contribute to tumor invasion and metastatic dissemination.

#### 3.2.3. TGF-β Signaling

TGF-β is a well-established mediator of radiation-induced stromal remodeling [21,49,50]. Radiation rapidly activates latent TGF-β within irradiated tissues, promoting fibroblast activation, myofibroblast differentiation, and extracellular-matrix remodeling [51,52]. Experimental studies have demonstrated that inhibition of TGF-β signaling reduces radiation-induced collagen III expression, supporting an important role for TGF-β in radiation-associated fibrotic responses [53]. Because TGF-β regulates the function of multiple stromal, epithelial, endothelial, and immune-cell populations, its biological effects are highly context-dependent [54]. These findings support an important role for TGF-β in coordinating radiation-induced stromal remodeling and fibrotic responses.

Collectively, radiation-induced stromal remodeling should not be regarded as a passive late toxicity but as a dynamic remodeling process involving reciprocal interactions among fibroblasts, immune cells, extracellular matrix, and soluble mediators. Persistent stromal activation can modify the structural and biochemical properties of irradiated tissues, potentially facilitating tumor-cell adhesion, migration, invasion, or survival. These stromal changes can interact with immune and vascular responses, further contributing to the maintenance of a remodeled tissue microenvironment.

### 3.3. Vascular Injury, Hypoxia, and Reparative Vasculogenesis

The vasculature is highly sensitive to ionizing radiation and represents one of the major targets of radiation-induced tissue remodeling [55,56]. Radiation damages endothelial cells, disrupts microvascular integrity, and impairs tissue perfusion, resulting in reduced oxygen and nutrient delivery to irradiated tissues [26]. Although vascular injury may initially restrict tumor growth by limiting blood supply, persistent microvascular dysfunction also creates hypoxic conditions that activate adaptive tissue-repair responses [26,57,58]. Consequently, radiation-induced vascular remodeling can have context-dependent effects, restricting tumor growth through impaired perfusion while, in some settings, generating hypoxic and reparative responses that favor recurrence or metastatic progression.

#### 3.3.1. Endothelial Injury and Hypoxic Adaptation

Endothelial cells play a central role in maintaining vascular homeostasis by regulating vessel integrity, tissue perfusion, leukocyte trafficking, and vascular permeability [59,60]. Radiation can induce endothelial apoptosis, vascular rarefaction, and prolonged impairment of microvascular function, which may contribute to tissue hypoxia [26]. Hypoxia subsequently activates multiple adaptive pathways that promote cell survival, tissue repair, angiogenesis, and extracellular-matrix remodeling [61,62]. Experimental studies support the biological consequences of this process. Tumors implanted into previously irradiated tissue can exhibit delayed primary growth associated with impaired vascular development while simultaneously displaying greater hypoxia and metastatic potential [25]. In melanoma xenograft models, radiation-induced hypoxia was associated with increased expression of IL-8 or uPAR, depending on the tumor model, and functional inhibition of these pathways reduced metastatic dissemination [25]. These findings support a mechanism in which hypoxia arising within a poorly vascularized irradiated tumor bed activates tumor-cell programs associated with angiogenesis, invasion, and metastatic dissemination.

#### 3.3.2. Reparative Vasculogenesis

Following substantial radiation-induced vascular damage, restoration of tumor vascular function may involve mechanisms distinct from conventional angiogenic sprouting. Rather than relying exclusively on sprouting of pre-existing vessels, irradiated tissues can recruit bone marrow-derived cells that participate in vascular repair, a process commonly referred to as vasculogenesis [26]. In irradiated tumors, hypoxia-induced chemokine signaling can promote recruitment of these cells and contribute to restoration of functional vasculature [26]. Evidence for this mechanism has been demonstrated most clearly in recurrent glioblastoma. Radiation-induced hypoxia increased HIF-1-dependent CXCL12 expression and promoted recruitment and retention of bone marrow-derived cells, predominantly CD11b^+^ myelomonocytic cells, through CXCL12–CXCR4 signaling [26]. Inhibition of HIF-1 or blockade of CXCL12–CXCR4 signaling prevented vasculogenesis and significantly delayed tumor recurrence [26]. These observations indicate that vascular repair following radiation may inadvertently support survival and regrowth of residual tumor cells.

#### 3.3.3. Vascular Remodeling and Metastatic Colonization

Radiation-induced changes in the vascular and perivascular microenvironment may also influence early steps of metastatic colonization. Radiation can alter paracrine signaling within irradiated tissues, thereby modifying tumor-cell adhesion, migration, and transendothelial migration [22]. Experimental models have demonstrated that irradiation of normal lungs before tumor-cell challenge increases pulmonary retention and subsequent metastatic colonization of circulating breast cancer cells [22]. CXCL12–CXCR4 signaling contributed to radiation-induced tumor-cell adhesion, migration, and transendothelial migration, and pharmacological inhibition of CXCR4 attenuated these effects [22]. Together with evidence from irradiated lung models demonstrating increased neutrophil recruitment and macrophage-dependent tissue remodeling [21,24], these findings suggest that vascular and perivascular alterations may act together with inflammatory and stromal responses to facilitate metastatic seeding.

Thus, radiation-induced vascular remodeling extends well beyond destruction of the tumor vasculature. Persistent endothelial injury, chronic hypoxia, and reparative vasculogenesis reshape the biological properties of irradiated tissues and interact closely with immune remodeling and fibrosis. These coordinated responses provide a mechanistic link between radiation-induced tissue repair and the establishment of tumor-promoting niches.

### 3.4. Radiation-Induced Cellular Senescence

Radiation-induced tissue remodeling can persist long after the initial radiation exposure. One mechanism that may contribute to this persistence is cellular senescence, a stress response characterized by prolonged cell-cycle arrest accompanied by continued metabolic and secretory activity [63,64]. Radiation can induce senescence in both malignant and non-malignant cells through persistent DNA-damage responses and other cellular stresses [65,66]. Although senescence can prevent the proliferation of damaged cells, senescent cells can remain within irradiated tissues and influence neighboring cells through their secretory activity [67,68].

A major feature of senescent cells is the senescence-associated secretory phenotype (SASP), which includes inflammatory cytokines, chemokines, growth factors, and matrix-remodeling factors [69,70]. The composition of the SASP varies according to cell type and tissue context, but its biological importance lies in the ability of senescent cells to modify the behavior of surrounding immune, stromal, and tumor cells [69,70]. Thus, radiation-induced senescence provides a potential mechanism through which a transient radiation injury can generate biological effects that persist beyond the period of acute tissue damage [67]. Direct experimental evidence for this mechanism has been obtained in the irradiated brain. Radiation-induced senescence of non-neoplastic cells within the brain microenvironment promoted subsequent glioblastoma growth in preclinical models [67]. More recent studies identified astrocytes as an important senescent population following cranial irradiation [67,68]. Senescent astrocytes promoted chemokine production and recruitment of peripheral myeloid inflammatory cells, generating an immune microenvironment that facilitated glioblastoma regrowth [68]. Together, these findings demonstrate that radiation-induced senescence in non-malignant cells can alter the surrounding tissue microenvironment and contribute to recurrent tumor growth.

Radiation can also induce senescence directly in surviving tumor cells. Therapy-induced senescent cancer cells remain metabolically active and can develop secretory programs capable of influencing neighboring malignant and non-malignant cells [71,72]. However, therapy-induced tumor-cell senescence is heterogeneous, and its phenotype and biological consequences vary according to cellular context and the nature of the therapeutic stress [73].

Thus, cellular senescence provides a biologically plausible mechanism linking transient radiation exposure with prolonged microenvironmental remodeling. Persistent senescent cells and their secreted factors can sustain communication among tumor, immune, and stromal compartments after the acute radiation response has subsided. In this context, senescence may contribute to the maintenance of radiation-induced tumor-promoting niches by prolonging inflammatory and tissue-remodeling signals that would otherwise resolve during normal tissue recovery.

## 4. Strategies to Prevent Radiation-Induced Tumor-Promoting Niches

**Figure 1:**
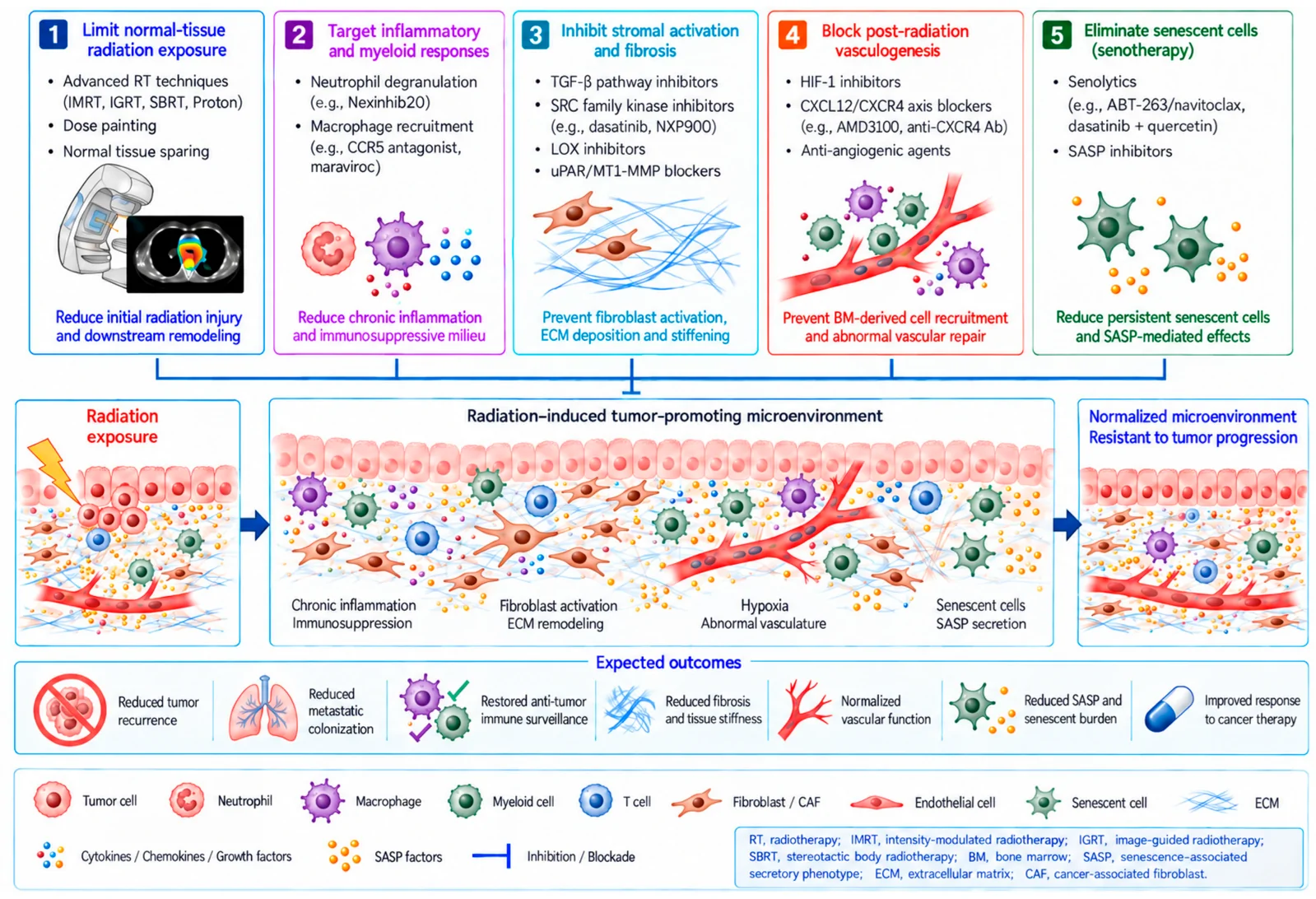
Strategies to prevent radiation-induced tumor-promoting niches. Schematic overview of potential strategies for preventing or attenuating the formation of tumor-promoting microenvironments following radiotherapy. Radiation exposure can induce persistent tissue remodeling characterized by chronic inflammation and immunosuppression, fibroblast activation and extracellular-matrix remodeling, hypoxia and abnormal vascular repair, and accumulation of senescent cells with senescence-associated secretory phenotype activity. Potential preventive approaches include (1) limiting unnecessary normal-tissue radiation exposure through advanced radiation-delivery techniques and normal-tissue sparing; (2) targeting radiation-induced inflammatory and myeloid responses, including neutrophil degranulation and macrophage recruitment; (3) inhibiting stromal activation and fibrotic remodeling; (4) blocking hypoxia-driven recruitment of bone marrow-derived cells and post-radiation vasculogenesis; and (5) eliminating persistent senescent cells or attenuating their secretory effects.

The experimental evidence described above indicates that several components of radiation-induced tumor-promoting niches are therapeutically modifiable. However, many of the inflammatory, stromal, and vascular responses triggered by radiation also contribute to normal tissue repair and recovery. Preventive strategies should therefore aim to limit persistent tumor-supportive remodeling while preserving the tumoricidal effects of RT and the physiological responses required for tissue restoration. Potential approaches include minimizing unnecessary normal-tissue exposure and, based on preclinical evidence, targeting radiation-induced inflammatory and fibrotic responses, post-radiation vasculogenesis, and persistent senescent cells (Figure 1).

### 4.1. Limiting Radiation Exposure to Normal Tissues

Because irradiation of normal tissues can initiate inflammatory, stromal, and vascular remodeling, reducing unnecessary normal-tissue exposure represents an upstream strategy for limiting the initial tissue injury that triggers these responses [74]. Modern radiation-delivery techniques, including intensity-modulated radiotherapy, image-guided radiotherapy, stereotactic approaches, and proton therapy, can improve dose conformity and reduce radiation exposure to surrounding normal tissues in appropriate clinical settings [74,75,76]. From the perspective of tumor-promoting niches, normal-tissue sparing could theoretically reduce the tissue injury that initiates subsequent microenvironmental remodeling. However, this possibility has not been directly demonstrated as a mechanism for reducing tumor recurrence or metastatic colonization in patients. Normal-tissue sparing is an established approach for reducing radiation toxicity and may also help limit tissue remodeling that could promote tumor recurrence or metastasis, although this additional benefit has not yet been demonstrated clinically.

### 4.2. Targeting Inflammatory and Stromal Remodeling

Preclinical studies provide direct evidence that specific inflammatory and stromal responses induced by radiation can be therapeutically targeted to reduce subsequent tumor growth. In previously irradiated lungs, neutrophil degranulation promoted regenerative Notch signaling within the irradiated tissue, which supported the growth of subsequently arriving breast cancer cells [24]. Pharmacological inhibition of neutrophil degranulation with Nexinhib20 reduced radiation-enhanced metastatic growth, and genetic deficiency of neutrophil elastase similarly impaired the metastatic phenotype [24]. These experiments demonstrate that specific functions of radiation-recruited neutrophils can be targeted to reduce metastatic colonization of previously irradiated tissues [24].

Macrophage recruitment represents another potential intervention point. In breast cancer models, macrophages accumulated within irradiated tissues before recruitment of circulating tumor cells, particularly when CD8^+^ T-cell surveillance was impaired [27]. Treatment with the CCR5 antagonist maraviroc reduced macrophage infiltration and decreased recruitment of circulating tumor cells to irradiated tissues [27]. These findings provide functional evidence that inhibition of radiation-associated macrophage recruitment can reduce the recruitment of circulating tumor cells to irradiated tissues under conditions of impaired adaptive immune surveillance [27].

Stromal remodeling can also be pharmacologically targeted. In irradiated lung and liver models, activation of SRC-family kinases (SFKs) in epithelial cells and fibroblasts was associated with macrophage-dependent fibrotic remodeling and formation of a tumor-permissive microenvironment [21]. Pharmacological SFK inhibition with dasatinib or NXP900 reduced myofibroblast activation and fibrosis and suppressed radiation-enhanced tumor-cell engraftment [21]. In tumor-bearing mice, combination treatment with RT and SFK inhibition also improved survival compared with RT alone [21]. These findings provide direct preclinical evidence that pharmacological modification of the radiation-remodeled host tissue can reduce its subsequent tumor-supportive properties [21].

Together, these studies demonstrate that radiation-induced inflammatory and stromal responses are experimentally modifiable. However, neutrophils, macrophages, and fibroblasts also participate in physiological tissue repair, and the consequences of broadly suppressing these populations during RT remain uncertain. Targeting specific tumor-supportive functions or signaling pathways may therefore provide a more selective strategy than broad suppression of the entire inflammatory or wound-healing response.

### 4.3. Targeting Post-Radiation Vasculogenesis

Radiation-induced vascular damage can generate hypoxic conditions that promote recruitment of bone marrow-derived cells and restoration of tumor vasculature through vasculogenesis [26]. This process provides another experimentally validated therapeutic target. In irradiated glioblastoma models, increased HIF-1 activity induced SDF-1/CXCL12 expression and promoted recruitment and retention of bone marrow-derived cells, predominantly CD11b^+^ myelomonocytic cells, through SDF-1–CXCR4 signaling [26]. Pharmacological inhibition of HIF-1 or blockade of the SDF-1–CXCR4 interaction reduced recruitment and retention of these cells, inhibited restoration of functional tumor vasculature, and prevented post-irradiation tumor regrowth [26]. Similar effects were observed with CXCR4-neutralizing antibodies [26]. These findings provide direct experimental evidence that inhibition of post-radiation vasculogenesis can suppress glioblastoma recurrence in preclinical models [26].

### 4.4. Targeting Radiation-Induced Cellular Senescence

Persistent senescent cells represent another potentially actionable component of the irradiated microenvironment. Radiation induces senescence in non-neoplastic cells, including astrocytes, which can remain metabolically active and maintain secretory activity capable of influencing neighboring tumor and immune cells [67,68]. These observations suggest that senescent cells may represent a therapeutic target for limiting persistent tumor-supportive remodeling after radiation exposure. Direct evidence for this strategy has been obtained in glioblastoma models. Cranial irradiation generated a senescent brain microenvironment that promoted subsequent glioblastoma growth, whereas pharmacological elimination of senescent cells with the senolytic agent ABT-263 reduced the tumor-promoting effects of the previously irradiated brain [67].

A more recent study demonstrated that radiation-induced senescent astrocytes promoted chemokine-dependent recruitment of peripheral myeloid inflammatory cells and facilitated glioblastoma regrowth [68]. Post-radiation treatment with senolytic regimens reduced the burden of senescent cells and prolonged survival in preclinical glioblastoma models [68]. Together, these studies provide direct evidence that elimination of persistent senescent cells after irradiation can suppress tumor-supportive effects of the irradiated brain microenvironment [67,68].

Whether senolytic strategies will have similar effects in other irradiated organs remains unknown. Radiation induces senescence in multiple cell populations, and both the composition of the senescence-associated secretory phenotype and its biological consequences vary according to cell type and tissue context [65]. Identification of the specific senescent populations that sustain tumor-promoting remodeling will therefore be important for broader application of this strategy.

### 4.5. Therapeutic Timing and Selective Targeting

A major consideration for all of these approaches is the timing of intervention. Many processes implicated in radiation-induced tumor-promoting niches—including innate immune recruitment, fibroblast activation, vascular repair, and cellular senescence—also have physiological functions following tissue injury [18]. Their complete suppression during RT could therefore interfere with normal tissue recovery or alter beneficial biological responses to radiation.

The available preclinical studies instead support the feasibility of targeting specific components of these responses [21,24,26,27,67,68]. Neutrophil degranulation, macrophage recruitment, SFK-dependent stromal remodeling, post-radiation vasculogenesis, and persistent senescent cells have each been experimentally manipulated to reduce tumor-promoting effects in defined models [21,24,26,27,67,68]. However, these studies do not yet establish a universal therapeutic window applicable across tissues or radiation regimens.

Future preventive strategies will therefore require definition of the temporal evolution of radiation-induced tissue remodeling and identification of biomarkers that distinguish resolving physiological repair from persistent tumor-supportive remodeling. Such information may enable interventions to target maladaptive host responses after RT while preserving tumor control, beneficial antitumor immune responses, and the physiological tissue-repair processes required for recovery.

## 5. Conclusions and Future Perspectives

Radiotherapy induces biological effects that extend beyond direct tumor-cell killing. Experimental evidence demonstrates that irradiated tissues can undergo persistent immune, stromal, extracellular-matrix, vascular, and cellular remodeling that influences residual or subsequently arriving tumor cells [19,20,21,22,23,24,25,26,27,28,29]. These interconnected responses suggest that radiation-induced tumor-promoting niches represent dynamic tissue states in which physiological repair processes become persistent or are exploited by tumor cells, potentially contributing to recurrence and metastatic colonization.

However, the clinical significance and generalizability of this phenomenon remain incompletely defined. Much of the causal evidence derives from preclinical models designed to isolate host-tissue effects from direct tumor irradiation [19,20,21,22,23,24,25,26,27,28,29]. Because radiation-induced tumor-promoting niches have not yet been specifically evaluated as a distinct clinical phenomenon, their incidence and attributable contribution to tumor recurrence or metastasis remain unknown, and the patient populations or clinical settings in which they may be most relevant have not been established. The resulting responses are likely to vary with radiation dose and fractionation, irradiated tissue, tumor type, and timing. Moreover, the apparent convergence of several mechanistic pathways and therapeutic targets may partly reflect the limited number and diversity of experimental models currently available, and their generalizability will require independent validation across additional tumor types, organs, radiation regimens, and experimental systems. Future studies should therefore define the spatial and temporal evolution of irradiated tissues using clinically relevant radiation regimens and determine which alterations are causally associated with subsequent tumor progression. Further studies specifically examining the temporal and causal relationships among these processes will be required to determine the extent to which they form a common interconnected tumor-promoting tissue state following irradiation.

Clinical validation will be particularly important. Integration of radiation-dose information with longitudinal imaging, tissue or liquid-biopsy analyses, and immune and molecular profiling may help determine whether persistent tissue remodeling predicts recurrence and identify biomarkers that distinguish physiological tissue repair from maladaptive tumor-supportive remodeling. Such biomarkers could ultimately guide the timing and selection of preventive interventions.

Beyond external-beam RT, an additional area that warrants investigation is whether radiation-induced tumor-promoting tissue remodeling can also occur following internal radiotherapy with therapeutic radiopharmaceuticals. Unlike external-beam RT, radiopharmaceutical therapy produces spatially and temporally heterogeneous radiation exposure according to the biodistribution, physiological uptake, retention, and clearance of the administered radiopharmaceutical, resulting in radiation exposure of non-target organs [77]. Normal-tissue injury following such exposure is well recognized, and experimental studies of ^177^Lu-labeled peptide receptor radionuclide therapy have demonstrated persistent molecular, functional, and morphological alterations in irradiated renal tissue [78]. However, direct evidence that these tissue alterations subsequently facilitate tumor-cell engraftment, recurrence, or metastatic colonization is currently lacking. Whether normal-tissue remodeling induced by therapeutic radiopharmaceuticals can generate tumor-promoting niches similar to those described following external-beam irradiation therefore represents an important question for future investigation.

Importantly, the concept of radiation-induced tumor-promoting niches should not be interpreted as evidence against radiotherapy. RT remains an essential and highly effective component of cancer treatment. Accordingly, the currently available evidence does not justify withholding, reducing, or otherwise modifying clinically indicated RT solely because of concern about radiation-induced tumor-promoting niches. Rather, recognition of these tissue responses identifies a potentially underappreciated consequence of radiation exposure and an opportunity to further improve long-term outcomes. Combining modern normal-tissue-sparing RT with selective targeting of persistent inflammatory, stromal, vascular, or senescent responses may ultimately enable durable tumor control while minimizing post-treatment tissue conditions that favor recurrence.

## Figures and Tables

**Table 1:** Principal preclinical models supporting radiation-induced tumor-promoting effects.

Study	Irradiated Tissue/Cell Type	Tumor Model	Radiation Regimen	Experimental Design	Proposed Mechanism	Tumor-Promoting Evidence	Causal Evidence
Barc ellos-Hoff and Ravani [19]	Mammary gland stroma	COMMA-D mammary epithelial cells	4 Gy, single dose	Normal mammary tissue irradiated before transplantation of non-irradiated COMMA-D cells	Radiation-induced stromal/microenvironmental remodeling	Increased tumor incidence, earlier tumor appearance, and increased tumor size	Non-irradiated epithelial cells were transplanted after irradiation; hemibody irradiation localized tumor promotion to the irradiated side
Bouchard et al. [20]	Mammary gland	D2A1 triple-negative breast cancer	6 Gy × 4 daily fractions	Mammary gland irradiated before implantation of D2A1 cells	MT1-MMP-dependent tumor-cell invasion within irradiated microenvironment	Increased local invasion, circulating tumor cells, and lung metastases	MT1-MMP knockdown abolished radiation-enhanced invasion, CTCs, and lung metastasis
Choi et al. [21]	Lung and liver	B16F10 melanoma, 4T1 breast cancer	22 Gy × 2 (days 1 and 3); additional lung model: 2 Gy × 10	Normal tissue irradiated before subsequent tumor-cell challenge	Macrophage recruitment/M2 polarization → fibrosis; SFK-dependent epithelial/fibroblast activation and stromal remodeling	Increased pulmonary tumor-cell engraftment and hepatic metastasis; reduced survival	Macrophage depletion reduced pulmonary engraftment; SFK inhibitors (NXP900/dasatinib) suppressed fibrosis and tumor-promoting effects; NXP900 also reduced radiation-enhanced liver metastasis
Feys et al. [22]	Lung/lung epithelial cells	4T1 and MDA-MB-231 breast cancer	10 Gy, single dose	Lung or lung epithelial cells irradiated before tumor challenge	Radiation-induced CXCL12/MIF secretion and CXCR4-dependent adhesion, migration, and transendothelial migration	Increased lung-specific metastatic colonization	CXCR4 inhibition suppressed radiation-induced metastasis-associated cellular activities; partial-lung irradiation spatially linked irradiation to enhanced metastasis
Milas et al. [23]	Prospective subcutaneous tumor bed	Ten syngeneic murine tumors (5 sarcomas, 5 carcinomas)	20 Gy, single dose	Tumor cells implanted into pre-irradiated versus non-irradiated tumor beds	Radiation injury to tumor-bed stroma (tumor-bed effect)	Slower primary tumor growth but increased metastatic spread in tumor-bed-effect models	Irradiation preceded tumor implantation, separating stromal irradiation from direct tumor-cell irradiation
Nolan et al. [24]	Lung	4T07 and 4T1 breast cancer; MMTV-PyMT-derived cells; Flo-1 esophageal adenocarcinoma; A549/H460 NSCLC	13 Gy, single dose; additional fractionated regimens: 4 Gy × 3 or × 4	Healthy lung irradiated before intravenous tumor-cell challenge	Activated neutrophils/degranulation → regenerative Notch signaling → increased cancer-cell stemness	Increased pulmonary metastatic colonization	Neutrophil depletion reduced metastatic burden; inhibition of neutrophil degranulation/Notch activation attenuated radiation-enhanced metastasis; targeted irradiation enriched metastases in the irradiated lung
Rofstad et al. [25]	Subcutaneous tumor bed	D-12 and R-18 human melanoma	10 or 20 Gy, single-dose	Human melanoma xenografts grown in pre-irradiated versus untreated beds	Impaired vascularization → hypoxia → IL-8 (D-12) or uPAR (R-18) upregulation	Increased pulmonary (D-12) or lymph-node (R-18) metastasis despite slower primary growth	Neutralization of IL-8 or uPAR reduced metastatic dissemination; dose-dependent tumor-bed effects
Kioi et al. [26]	Intracranial glioblastoma/tumor vasculature	Human glioblastoma xenografts (principally U251)	15 Gy, single dose; or 2 Gy × 5 daily fractions	Established intracranial tumors irradiated and followed for recurrence	HIF-1 → SDF-1/CXCL12–CXCR4 → recruitment of CD11b^+^ bone-marrow-derived cells → vasculogenesis	Restoration of functional vasculature and post-irradiation tumor regrowth	HIF-1 inhibition, CXCR4 blockade/neutralization, and inhibition of BM-derived-cell recruitment impaired vascular restoration and prevented recurrence
Rafat et al. [27]	Mammary fat pad and surrounding irradiated tissues	4T1 and MDA-MB-231 breast cancer	20 Gy, single dose	Normal tissue irradiated in mice bearing a tumor at a distant/contralateral site; recruitment of circulating tumor cells assessed	CD8^+^ T-cell deficiency → excessive macrophage recruitment; CCL3/4/5–CCR5 signaling → tumor-cell recruitment	Preferential recruitment/reseeding of circulating tumor cells into irradiated tissues	Tumor recruitment localized to irradiated tissues; macrophage accumulation preceded tumor-cell recruitment; CCR5 antagonist maraviroc reduced macrophage and tumor-cell recruitment
Shen et al. [28]	Tumor cells/A549 tumor xenografts	A549 human lung adenocarcinoma	In vitro: 0–10 Gy; xenografts: 10 Gy × 1 or × 2 (12 h interval)	Irradiated tumor-cell conditioned medium tested on non-irradiated tumor cells; locally irradiated xenografts examined for LOX	Radiation-induced secretion of enzymatically active LOX	Increased invasiveness of non-irradiated tumor cells	LOX knockdown in irradiated cells abolished the invasion-promoting activity of conditioned medium
Li et al. [29]	Pancreatic carcinoma-associated fibroblasts (CAFs)	PANC-1 and MIA PaCa-2 pancreatic cancer cells	4 Gy, single dose to CAFs	Tumor cells exposed to irradiated CAFs/conditioned medium; preconditioned tumor cells assessed in experimental lung-metastasis model	CAF-derived CXCL12 → CXCR4/p38 signaling → EMT and invasion	Increased migration/invasion, EMT, and lung metastatic growth of preconditioned tumor cells	CXCR4 blockade with AMD3100 reduced EMT/invasion and lung metastasis associated with irradiated-CAF conditioning

**Table 2:** Clinical studies reporting outcomes relevant to the hypothesis of radiation-induced tumor-promoting niches.

Study	Study Design and Population	Clinical Phenomenon Relevant to the Proposed Niche	Quantitative Findings	Statistical Analysis/Adjustment	Major Limitations and Interpretation
Dao and Kovaric, 1962 [30]	Historical observational study of 354 women with breast cancer treated with radical or simple mastectomy, with or without postoperative irradiation	Altered pulmonary and skin metastatic patterns after postoperative irradiation	Among clinically and pathologically comparable patients undergoing radical mastectomy, ipsilateral pulmonary metastases were reported in 14% without irradiation versus 37% with postoperative irradiation; skin metastases occurred in 34% versus 52%, respectively	No adjusted effect estimate, CI, or *p* value was reported in the accessible abstract	Non-randomized treatment allocation; small irradiated radical-mastectomy subgroup (*n* = 30); historical staging, systemic therapy, and radiation techniques. The study suggests an altered metastatic pattern but cannot establish causality or a radiation-induced niche mechanism
PORT Meta-analysis Trialists Group, 1998; Burdett and Stewart, 2005 [31,32]	Individual-patient-data meta-analysis of randomized trials of PORT versus surgery alone after complete resection of NSCLC; original analysis included 2128 patients from 9 randomized trials	Recurrence-related outcomes following postoperative irradiation	The original analysis showed an adverse effect on OS (HR, 1.21; 95% CI, 1.08–1.34) and an absolute 7% reduction in 2-year OS (55% to 48%). The updated analysis reported similar unfavorable effects on local recurrence-free, distant recurrence-free, and overall recurrence-free survival, but study-specific numerical effect estimates for these recurrence endpoints were not provided in the PubMed abstract	Individual-patient-data, intention-to-treat meta-analysis of randomized trials	Randomization limits treatment-selection bias, but the trials largely used historical RT techniques. Neither analysis assessed irradiated-tissue remodeling or niche-related biomarkers; therefore, recurrence or survival outcomes cannot be mechanistically attributed to a tumor-promoting niche
Sebastian et al., 2020 [33]	Single-institution retrospective study of 554 patients with cT1–2N0 NSCLC treated with lobectomy (*n* = 389) or SBRT (*n* = 165); propensity-score-matched cohort: 132 SBRT and 85 lobectomy patients	Local, regional, and distant recurrence after SBRT versus surgical resection	Local recurrence: HR, 6.80 (95% CI, 1.92–24.10; *p* = 0.003); regional nodal recurrence: HR, 2.58 (95% CI, 1.17–5.68; *p* = 0.018); distant recurrence: HR, 1.19 (95% CI, 0.57–2.52; *p* = 0.64)	Cox proportional-hazards analysis with nearest-neighbor propensity-score matching to reduce measured confounding	Propensity matching reduces but does not eliminate residual confounding or treatment-selection bias. Differences in recurrence cannot distinguish host-tissue remodeling from differences in tumor eradication, staging, treatment modality, or baseline patient characteristics
Woodward et al., 2010 [34]	Analysis of two breast cancer datasets: 229 LRR cases among 1500 patients enrolled in prospective MDA studies and 66 LRR cases among 318 patients in a randomized BCCA PMRT trial	Whether recurrence arising after prior irradiation exhibits a more aggressive clinical phenotype	In the MDA cohort, prior PMRT was initially associated with poorer survival after any LRR, but PMRT-treated patients had more advanced baseline disease and more frequent distant metastasis before LRR (63% vs. 34%; *p* = 0.005). Among patients with isolated LRR, OS did not differ by prior PMRT (*p* = 0.33). In the BCCA randomized dataset, DM-free survival after any or isolated LRR did not differ according to prior PMRT (*p* = 0.75 and *p* = 0.26), nor did OS after any or isolated LRR (*p* = 0.93 and *p* = 0.28)	Comparison within prospective MDA datasets and an independent randomized PMRT trial dataset; analyses stratified by recurrence context	Provides important counter-evidence: prior irradiation was not associated with a demonstrably more aggressive recurrence phenotype after accounting for major baseline differences. The analysis nevertheless evaluates clinical recurrence behavior rather than tissue-remodeling mechanisms

## Data Availability

No new data were created or analyzed in this study. Data sharing is not applicable to this article.

## Abbreviations

CCR5, C-C chemokine receptor type 5; CXCL12, C-X-C motif chemokine ligand 12; CXCR4, C-X-C motif chemokine receptor 4; DAMPs, damage-associated molecular patterns; ECM, extracellular matrix; EMT, epithelial-to-mesenchymal transition; HIF-1, hypoxia-inducible factor 1; IGRT, image-guided radiotherapy; IL-8, interleukin-8; IMRT, intensity-modulated radiotherapy; LOX, lysyl oxidase; MMPs, matrix metalloproteinases; MT1-MMP, membrane-type 1 matrix metalloproteinase; ROS, reactive oxygen species; RT, radiotherapy; SASP, senescence-associated secretory phenotype; SBRT, stereotactic body radiotherapy; SDF-1, stromal cell-derived factor 1; SFKs, SRC-family kinases; TGF-β, transforming growth factor beta; uPAR, urokinase-type plasminogen activator receptor.
